# Evaluating the couriers’ experiences of logistics platform: The extension of expectation confirmation model and technology acceptance model

**DOI:** 10.3389/fpsyg.2022.998482

**Published:** 2022-09-27

**Authors:** Qiumei Zheng, Chenglong Li, Shizhen Bai

**Affiliations:** Harbin University of Commerce, Harbin, China

**Keywords:** online logistics platform, technology acceptance model, information system quality, user interface, knowledge sharing motivation, expectation confirmation model, perceived value

## Abstract

The current study integrated the Extended Technology Acceptance Model (TAM) and included information system quality (ISQ), user interface (UI), knowledge sharing motivation (KSM), the expectation confirmation model (ECM), safety management practices (SMP), interface aesthetics (IA), and perceived value (PV) to evaluate the logistics couriers’ experience while using an Online logistics platform. This research examines the relationships of KSM, SMP, and ISQ on the TAM’s, perceived usefulness (PU), and perceived ease of use (PEOU). In addition, it explores the relationship between UI on PEOU. Furthermore, to explore the impact of ECM, it examines the impact of confirmation (CON) on PU and satisfaction (SAT). Finally, this research explores the impact of logistics couriers’ SAT on continuous intention (CI). According to the findings of this study, UI did not have a significant association with PEOU. Furthermore, KSM was found to significantly impact PEOU, while having no significant effect on PU. Moreover, SMP was found to have no significance on PEOU, however, SMP was discovered to be in a significant association with PU. In addition, ISQ was found to significantly impact PEOU, PU, and, PV. Moreover, CON was in a significant relationship with PU, while not having a significant impact on SAT. Furthermore, IA did not significantly impact PV. Also, SAT was significantly impacted by PU, while not having any significant impact from PEOU, and PV. Besides, PEOU was discovered to significantly impact PU. Finally, SAT was found to be in a significant relationship with CI.

## Introduction

COVID-19, a severe acute respiratory virus, has landed in many nations throughout the world. The United States Department of Health and Human Services and the World Health Organization (WHO) announced COVID-19 as a pandemic because it has been spread globally affecting most people. The day-to-day lives of the people have been changed due to this pandemic. An increasing number of people have started ordering online through online logistics platforms (OLPs) because of the evolution of COVID-19 and its contagious nature, taking into account the transmission of this disease on the streets. Over time, however, the collection of the order by the customer from the representative of the courier service has become problematic. It looks like a challenging situation while delivering online orders. These circumstances in the pandemic create unpredictability in the delivery of online orders (Yaprak et al., 2021). The abstracts of user interface (UI) have become a subject of keen interest within the discipline of human–computer interface (HCI) and particularly in the field of research related to user experience. The increased aesthetic factors apart from conventional usability contribute to all aspects of user satisfaction (Hartmann et al., 2007) and the total credibility of the product and the system (Norman, 2004; Seckler et al., 2015). Moreover, several types of research revealed the effects of aesthetics on perceived usability (Phillips and Chaparro, 2009), on test cases of usability (Sonderegger and Sauer, 2010), on trustworthiness and integrity (Robins and Holmes, 2008), and enjoyment and fun (Seckler et al., 2015). Online services and internet infrastructure influences the development of electronic commerce (Ponte et al., 2015). The role of conventional communications, for example, the telephone, has diminished in favor of a new model of communication that uses the internet, websites, e-mail, social media, and e-travel (Shih-Chih and Shing-Han, 2010). Customers and service providers no longer need to be physically present for transactions to occur over virtual channels in these high-tech settings. The tendency is to move toward online services from person-to-person contact (Shih-Chih and Shing-Han, 2010; Breidbach et al., 2013; Masri et al., 2020).

Organizations have moderately increased their capital in information technology (IT) for planning to boost the productivity of their company operations, support the business decision, and raise the efficiency and their business processes. Thus, organizations are using IT strategically to gain competitive advantages. Because of this, the majority of the research concerning the adoption and spread of technology has been carried out by researchers and professionals working in the hospitality industry (Masri et al., 2020). Considering the provisioning of benefits from IT, it would appear that an inevitable trend is developing, which is the widespread usage of technology (Ham et al., 2005). Despite the increased usage of technology, several studies have been initiated to analyze the interaction between external factors and the technology acceptance model (TAM) paradigm to explain OLPs adoption behavior. Additionally, due to the complicated process involving both external and internal variables as well as their specific characteristics, technology acceptance in OLPs requires distinctive methodologies in analyzing technology adoption behavior (Wang and Qualls, 2007; Kim et al., 2008). Studying previous research (Wu, 2013; Mohammadi, 2015; Alsabawy et al., 2016; Hart et al., 2019; Al-Fraihat et al., 2020; Pal and Vanijja, 2020; Prasetyo et al., 2021), the Delone and McLean IS Model could use behavioral intentions to measure how a system is used, and the UI is a preliminary of perceived ease of use (PEOU) that decides how users interact with each other (Mohammadi, 2015; Hart et al., 2019; Prasetyo et al., 2021). Therefore, the objective of this study is to find out how UI will impact the PEOU of TAM.

Many organizations are interested in and paying attention to issues related to knowledge-sharing motivation (KSM). Within businesses, the medium of trade is rarely monetary, however, several different currencies are accepted that drive the market for knowledge (Davenport and Prusak, 1998). People who are willing to offer their expertise do so because doing so allows them to receive the rewards of distinction, reciprocation, and benevolence. Based on their points of view, this study focuses on the social exchange theory (SET) based on motivations for sharing knowledge, such as distinction, reciprocation, and benevolence. Distinction and reciprocation are examples of external inspiration, whereas benevolence is an example of internal aspiration. The distinction is perceived as an illustration of external inspiration given that it is referred to as ‘Reputation Design’, which is a type of system attribute (Hung et al., 2011). However, Davis’s (1993) TAM, which highlights PEOU and perceived usefulness (PU) as the most crucial variables in the acceptability of an IT system, is the most influential model on employee acceptance of knowledge sharing in organizations. It is presumed that the influencing variables determining the acceptability of new IT will vary depending on the context, target users, and technology. Therefore, today’s research aims to analyze the correlation between TAM and KSM (Hung et al., 2011). Moreover, safety management is considered an important entity of overall business management and is implemented by using a variety of Safety Management approaches (Vinodkumar and Bhasi, 2010). It was proposed that safety management practices (SMP) and TAM could explain the reason workers prefer to use OLPs. The usefulness and flexibility of the TAM in describing the adoption of various technologies led to its incorporation as the variable in this relationship. Hence, this research evaluates the impact of SMP on TAM (Wong et al., 2021). About an information system’s critical success elements, the value of an information system ought to be evaluated in three aspects: system, quality of service, and information (Pitt et al., 1995; Kim et al., 2008). This research analyzes the influence of the three features of information system quality (ISQ) on TAM (Kim et al., 2008; Prasetyo et al., 2021).

In addition, the expectation confirmation model (ECM) endorses the two important aspects of confirmation (initial-adoption attitude) and PU (post-adoption attitude), which are reciprocally related to satisfaction and continued intent. Moreover, confirmation also indicates a connection to PU. Confirmation of performance is evidence of the anticipated and perceived effectiveness of using online platforms (Halilovic and Cicic, 2013). PU could be defined as, the extent to which the particular product or equipment is highly applicable for completing tasks. Logistics couriers would expect an OLPs’ PU to have increased confirmation (Elwalda et al., 2016; Zhao and Khan, 2021). Furthermore, the satisfaction of logistics couriers is a crucial component in the propensity to participate in OLPs. Moreover, flow experience and fulfillment are regarded as the emotional and logical perspectives of users who use online platforms. Thus, a substantial link exists between these factors (Rose et al., 2012; Foroudi et al., 2018; Sharma and Sharma, 2019; Zhao and Khan, 2021). Therefore, the objective of this research is to ascertain the impacts of confirmation on logistics couriers’ satisfaction and PU.

Aesthetics is considered a non-instrumental feature that plays a substantial role in the appeal and use of an item (Hassenzahl and Tractinsky, 2006). From the users’ standpoint, this type of experience is substantial in shaping the user’s inclination and usage commitments (Hong et al., 2017). An earlier study suggests that aesthetically alluring designs of the UI are much more convenient to use, therefore highlighting the significance of aesthetics in interface design (Kurosu and Kashimura, 1995). Researchers developed a measurement technique that spans a greater range of interface aesthetics (IA) construct perceptions (Moshagen and Thielsch, 2010; Wang and Hsu, 2019). Their evaluation includes four distinct facets of user-focused aesthetic perception which include colorfulness, craftsmanship, diversity, and simplicity (Wang and Hsu, 2019). On the other side, the idea of value has continuously arisen from many consumer behavior studies (Gallarza and Saura, 2006; Kuo et al., 2009; Masri et al., 2020). Previous research has advised that perceived value (PV) is a more important determinant of repurchase intention than contentment, trust, or allegiance (Gallarza and Saura, 2006; Masri et al., 2020). In this study, the interrelationship of ISQ and PV (Masri et al., 2020) as well as the correlation between IA and PV (Wang and Hsu, 2019) will be examined. The effects of PV (Masri et al., 2020), PU (Zhao and Khan, 2021), and PEOU (Tawafak et al., 2018b), on logistics courier satisfaction, will also be evaluated in this study. Additionally, this research will find the effects of PU on PEOU (Zhao and Khan, 2021). Lastly, this study will determine the consequences of contentment on ongoing intention (Zhao and Khan, 2021).

This study is unique from the previous studies because it integrated the Extended Technology Acceptance Model (TAM) and included ISQ, UI, knowledge sharing motivation (KSM), the ECM, safety management practices (SMP), IA and perceived value (PV) to evaluate the logistics couriers’ experience while using an Online logistics platform. This study addresses the following research gaps in evaluating the logistics courier’s experience with OLPs: This study first investigates the correlations between KSM, SMP, and ISQ and TAM’s PU and PEOU. The second objective of this analysis is to study the effect of ECM; hence, it investigates the impact of confirmation on PU and satisfaction. Thirdly, this research investigates the effect of ISQ and IA on PV. Fourthly, this research investigates the effects of PU, PEOU, and PV on satisfaction. Fifthly, this study investigates the impact of PU on PEOU. Lastly, this study investigates the relationship between logistics courier pleasure and continued intention. In addition, this study has significant practical and theoretical findings. These data can be utilized by practitioners and managers as guides for the development of online portals, operations, and user assistance. The assessment of ISQ can improve goods and services, enabling managers and enterprises to produce superior commodities in the OLP. This research study’s observations not only reflect a perspective on ISQ improvement but also on enhancing user relationship value via the OLP provider’s application of customer contentment.

## Theoretical background

### Interface aesthetics

The significance of IA originates from Kurosu and Kashimura’s (1995) findings, suggesting that interface designs that visually appeal are more convenient to use. The aesthetics of UIs have emerged as an important subject of discussion in the field of interactive product design or human-computer interaction. These aesthetics are not only limited to visuals, but a pleasing experience is something that the IA provide users (Tuch et al., 2012). Adding to the IA on electronic media gadgets strengthens a greater span of user adoption (Wang and Hsu, 2019). Kim et al. (2003) studied the UIs of websites and outlined the typical aesthetic design components and the emotional responses toward them by the users (Kim et al., 2003). Research conducted in the past suggested that mobile interfaces formulated on visual aesthetics are an ideal consolidation of function, aesthetics, and content (Wang and Hsu, 2019). Mobile UIs incorporate a composition of design aspects namely color, text, graphics, etc., to offer intuitive perception to users. Simultaneously, aesthetics and interface visualization are intuitive and acknowledged as beauty throughout the user experience. To demonstrate user satisfaction and happiness, the significant determinant is the visual aesthetics of user-computer interfaces (Choi and Lee, 2012). At the moment, numerous constructs related to the aesthetics of UI have been pointed out in empirical research, which includes diversity, uniformity, visual complexity, and colorfulness of UI (Wang and Hsu, 2019). Moshagen and Thielsch (2010) instigate an additional evaluation process that incorporates a wider impression of constructs related to IAs. The analysis consists of 18 items, out of those, four various factors of user-oriented aesthetic impression are determined: simplicity, diversity, colorfulness, and craftsmanship. Simplicity is highly relevant to Lavie’s and Tractinsky (2004) classical aesthetics, whereas diversity is highly associated with Lavie’s and Tractinsky (2004) expressive aesthetics. Moreover, two dimensions that are relatively new craftsmanship and colorfulness were also originated. Colorfulness incorporates constructs relevant to the measurements of colors, its factors, craftsmanship contemplates the correlated interface design, and the utilization of today’s technology (Moshagen and Thielsch, 2010). In this research, the authors also examine IA by utilizing four various factors of the user perceptions (colorfulness, diversity, simplicity, and craftsmanship). It could accurately examine IAs and is more responsive to design features of aesthetic perception related to color (Seckler et al., 2015; Wang and Hsu, 2019).

### Information system quality

DeLone and McLean (1992) proposed the information system. The study presented the model, which is comprised of three considerable dimensions: information quality, user satisfaction, and system quality in the framework of business organizations. After 10 years, in an improvement to the initial Information System literature, the researchers incorporated quality of service into the ISM to evaluate the system based on seven parameters: information quality, service quality, system quality, information use, user satisfaction, use and net benefits (DeLone and McLean, 2003; Masri et al., 2020). This generates a distinct definition of the information system (Chung and Koo, 2015). The system quality incorporates response time, system availability, and system reliability (DeLone and McLean, 2003; Dedeke, 2016). Quality of service is determined by the user’s impressions of the performance of service (Wang, 2015). The quality of service quantifies the difference between what the users’ desires and wants are and what is provided to meet those desires (Dedeke, 2016). As an example, the quality of service on multi-communication structures enables users’ complaints to be promptly addressed. Regarding information quality, user-perceived efficacy of the quality of system assessments has included accuracy, adequacy, relevance, quality, timeliness, and sequencing (Hosseini et al., 2016). To embrace sophisticated technology in the OLP environment, this research paper constructs three dimensions of service quality, system quality, and information quality as ISQ (Masri et al., 2020).

### Technology acceptance model

Davis (1993) introduced the TAM which is based on aspects of social psychology and the theory of innovation diffusion. It provides a useful tool for examining the interactivity and acceptability of thoughts and novelties. The TAM employs two approaches, like the PEOU and PU to determine the ultimate preference of users when examining the users’ motivation for recognizing (or rejecting) the most recent technological discoveries. The framework has been widely used to analyze user reactions in a variety of research domains (Do et al., 2020; Xu et al., 2021). Using the TAM, which integrates two main concepts, for instance, PEOU (Besbes et al., 2016), the web-based application’s usage and recognition have been extensively studied. The theory of reasoned action and information systems suggests that PEOU and PU are essential determinants of the intention to implement online platform services (Hyun et al., 2021; Xu et al., 2021). It shows that these two essential TAM principles significantly impact OLP’s acceptance intention (Xu et al., 2021). Davis (1989) concluded that the TAM’s external variables can influence the perceptions of PU and PEOU. In this manner, numerous studies have confirmed the external characteristics of a TAM (Amoako-Gyampah and Salam, 2004; Burton-Jones and Hubona, 2006; Kim et al., 2008). These findings imply that external factors such as personal qualities, system features, and organizational factors were significant in predicting technology acceptance. Additionally, it is believed that the perceived value (PV) of a system has a substantial effect on the positive attitude about usage (Kim et al., 2008). As external variables of TAM, this study examines the quality of information system KSM, SMP, and PV variables.

### The expectation confirmation model

Bhattacherjee (2001) proposed the ECM that integrates PU and expectation confirmation theory (ECT) to investigate the intention of continuing usage within the domain of information systems. A logistics courier’s continuous intention to use an information system is typically tied to the rationale at the back of the logistics courier’s reuse purpose in ECT, according to Bhattacherjee (2001). Commonly, to examine the application of design structures via online platforms, ECM has been employed (Lu et al., 2019). Particularly, ECM suggests two important influencers of PU and confirmation that are jointly associated with satisfaction and the intent to implement the OLP continuously. In addition, confirmation indicates a relationship to PU. The confirmation describes the relationship of the expected effectiveness with the perceived effectiveness of online platforms (Halilovic and Cicic, 2013). On the other hand, PU relates to an object’s effectiveness in completing a specific task. To assure the usefulness of digital platforms, users may improve their performance levels (Elwalda et al., 2016). To participate in online actions, satisfaction is also viewed as a crucial element (Wu et al., 2020; Zhao and Khan, 2021). Thong et al. (2006) discuss several verifiable ECM studies that were utilized to explore continuous information technology usage behavior. They contend that user experiences have a substantial effect on post-adoption and indicate IT or IS usage, making them a requirement for interpreting sustained IT or IS usage behavior. Chou et al. (2010) utilized ECM to comprehend the intention of continual knowledge generation in online communities. They explain that different factors such as socialization, externalization, etc. had a significant impact on the performance evaluation of the intention to continue knowledge creation. Baker-Eveleth and Stone (2015) investigated the extent to which jobseekers’ website experiences confirm or disprove their company-related assumptions. They also investigated the extent to which these expectations affect their intentions, satisfaction, perceived usefulness, and expectation confirmation, and how these factors directly or indirectly influence user intention on the website (Hariguna et al., 2017).

### Social exchange theory and knowledge-sharing motivations

Social exchange theory advocates ‘cost minimization and benefit maximization’, this frequent axiom encapsulates most of the knowledge buried in the social exchange process, wherein the individual motives are classified into extrinsic and intrinsic benefits (Deci and Ryan, 1980), that are essential for knowledge transfer (Hung et al., 2011). Extrinsic motivation refers to the idea when an individual is motivated extrinsically, his or her behavior will be to acquire some tangible benefit or desirable outcome (Deci and Ryan, 1980), such as organizational incentives. These incentives may include a bonus or compensation for giving knowledge (Kankanhalli et al., 2005; Hung et al., 2011), and reputational rewards to boost his or her reputation and social status in the organization (He and Wei, 2009; Hung et al., 2011). It can also be a form of knowledge that is contributed due to the cooperation individuals obtain from each other (Kankanhalli et al., 2005; He and Wei, 2009). Intrinsic motivation refers to the idea that, when an individual is motivated intrinsically, his or her behavior will be to participate in a task specifically for the satisfaction or contentment that is gained from participating in that activity (Deci and Ryan, 1980). Some illustrations of intrinsic motivation are when individuals attain pleasure through knowledge-sharing, and they enjoy doing that just because they like helping others (Kollock, 1999; Lin, 2007). Those individuals with knowledge self-efficacy believe that they can provide knowledge that is valuable and are ready to contribute to others (Kankanhalli et al., 2005; Lin, 2007; Hung et al., 2011) and self-worth gained from knowledge-sharing (Bock et al., 2005). Reciprocity, altruism, and reputation are the three motivational variables underlying this study (Hung et al., 2011).

## Hypothesis development

### User interface and perceived ease of use

The most dominant factor of an online meeting platform persists to be an engaging portal with numerous users. These practices encouraged learners to continue utilizing online meeting platforms (Hart et al., 2019; Prasetyo et al., 2021). As per Mohammadi (2015), the UI is where a user gains control of a system or technology. Additionally, it was discovered that the use of the features attracts students. The features would ultimately increase the user’s attractiveness and decrease the response time required for the contents to completely load (Mohammadi, 2015; Prasetyo et al., 2021). An effective interfaced system ought to be simple to operate (Cho et al., 2009). Poorly illustrated icons and buttons, for example, can cause uncertainty and misinterpretation. In comparison, a well-designed and organized screen can facilitate easier screening and identification of pertinent information (Thong et al., 2006; Cho et al., 2009). Moreover, the mouse interface simplifies system control to a series of clicks. Undoubtedly, a simple UI will lessen the struggle required to utilize a system. It will allow people to operate the system without difficulty. Additionally stated that UIs ought to be easy to operate and should be perceived as easy to operate. Whereas, PEOU is described here as the extent to which a prospective user anticipates the e-learning system which is self-paced to be effortless (Davis, 1989; Cho et al., 2009). If an interface is user-friendly to operate but isn’t recognized as easy, it will be considered as difficult to use, and it will not be easy for the user to adopt the system (Ozen and Basoglu, 2007; Cho et al., 2009). Graphical user interfaces (GUIs) are widely used for desktops, partially due to the idea that they are simple to use. PUID may therefore have a favorable influence on PEOU (Cho et al., 2009).

Consequently, the corresponding hypothesis is proposed.

Hypothesis 1: The user interface has a substantial impact on PEOU.

### Knowledge sharing motivation and technology acceptance model

System characteristics are acknowledged as an external variables’ set, which can impact PEOU and PU (Davis, 1989; Hung et al., 2011). System features are acknowledged as an external variable of TAM. There are substantial connections between system variables and TAM components that have been identified. Additionally, PU is influenced by image, or social status, which is enhanced through the use of technological innovations. The enhanced influence and power coming from an elevated position offer a basic foundation to increase output and efficiency (Venkatesh and Davis, 2000; Hung et al., 2011). Consequently, a person may believe that utilizing the OLP will enhance their work performance. A person may therefore view the system as beneficial because it enhances their reputation and credibility. Individuals might put more effort into both extrinsic and intrinsic motivations. According to a previous study, reputation development is a powerful motivator to engage in electronic systems of practice. Supposedly, user sharing motivation depends on extrinsic reciprocity, such as the anticipation of possible collaboration cooperation and the presumption that essential knowledge will be offered, resulting in self-belief that work output will enhance. Then, users with reciprocating motives will perceive that the OLP may increase the speed and effectiveness of KSM, fostering the idea that the system is valuable (Hung et al., 2011). Therefore, an individual’s higher standard of reciprocation would result in stronger PU due to increased confidence in the idea that the required knowledge would be reciprocated favorably. According to Malhotra and Galletta (2004), individual motivations can positively impact PU, PEOU, and CI. This research considers altruism as the perceived enjoyment derived from assisting others by giving knowledge via an online platform (Hung et al., 2011). Through internet platforms, altruistic individuals would intend to communicate their experiences with others (Kankanhalli et al., 2005; Hung et al., 2011). They also experience greater satisfaction, which stems from the intrinsic amusement of helping others (Krebs, 1975; Constant et al., 1996). People with greater altruism, according to Hsu and Lin (2008), are willing to improve the well-being of others. If a user has a high level of altruism as a result of his or her enthusiasm and enjoyment in supporting everyone else by transfer of knowledge via OLP, he or she will be capable to dissipate the necessary power to tackle all the obstacles while using OLP. Thus, the web platform will become considerably more user-friendly (Hung et al., 2011). Consequently, the following hypotheses can be proposed:

Hypothesis 2: KSM has a significant impact on PEOU.

Hypothesis 3: KSM has a significant impact on PU.

### Safety management practices and technology acceptance model

Safety management is a subsystem of overall organizational management and is executed using a variety of safety management techniques (Vinodkumar and Bhasi, 2010; Wong et al., 2021). Safety management techniques, such as SOPS (safety-offense points), SS (safety supervision), and ST (safety training), are significant determinants of employees’ OLP utilization (Wong et al., 2020). Through the implementation of a SOPS, employees who conduct safety offenses will be given safety offense points. Employees who accumulate predetermined safety offense points would be held accountable and may get unemployed for a predetermined amount of time. The SOPS concept originated via the penalty point system (PPS) in transportation safety. PPS was implemented to curb drivers’ risky driving behavior (Gras et al., 2014). SS measures safety performance (of employees) in the construction as opposed to predefined standards. A proactive technique to preventing harmful employee behavior and an indicator highlighting the significance of workplace safety are also included. Effective SS may display management’s dedication toward safety (Wong et al., 2021). ST is a technique for training construction employees with safety knowledge and is crucial for the success of projects (Guo et al., 2017). Particularly, staff can understand to detect safety threats and implement the appropriate safety measures to mitigate them. ST is essential for improving staff safety (Bhandari et al., 2019). Nonetheless, no research has investigated the effects of SS, ST, and SOPS on OLPs. Although prior research has not examined the effects, it is hypothesized that these SMP have a good influence on PU and PEOU (Wong et al., 2020, 2021). Consequently, the following hypotheses can be proposed:

Hypothesis 4: SMP has a significant impact on PEOU.

Hypothesis 5: SMP has a significant impact on PU.

### Information system quality and technology acceptance model

According to DeLone and McLean (2003), information quality is the most important factor in an ISQ’s success. According to academics, ISQ influences PEOU and PU (Igbaria et al., 1995; Venkatesh and Davis, 1996; Agarwal and Prasad, 1999; Kim et al., 2008). In defining the performance of information and the OLP environment, information quality is a crucial and critical component. The information plays a crucial role in accomplishing learning goals and overcoming the severe obstacles caused by poor information quality (Alsabawy et al., 2016). Furthermore, Wu (2013) found a substantial link between information quality and PU. Therefore, this research paper may conclude that improved information quality throughout the OLP environment can result in an increase in the proportion of PU (Prasetyo et al., 2021). The relationship between system quality and PEOU was not empirically demonstrated, according to Al-Fraihat et al. (2020). It is discussed that the absence of system quality support frequently has a direct impact on the system and the users’ comprehension of an OLP. The PEOU ought to be examined for platform utilization. Moreover, given the available communication methods such as email, messages, and forums, data from forums and messages might communicate personal information and data that a user might not want to be available to the external world via ISPs. Therefore, providing information prior to their use of the system or technology will increase their comprehension and dramatically alter their perceptions toward the overall usefulness of the OLP (Al-Fraihat et al., 2020; Prasetyo et al., 2021). Additionally, system quality is essential to user beliefs (Kim et al., 2008). Similarly, Ruth (2000) conducted research to find the impact of system quality on PEOU and Internet shopping. The results indicated that system quality had no significant impact on Internet shopping behavior, whereas PEOU and PU were comparatively strong and positive, and their effects are anticipated to be direct and constant. Ahn et al. (2004) categorized the quality of an Internet shopping mall’s online properties as a system, service quality, information quality, and system quality. ISQ was found to influence PEOU and PU (Kim et al., 2008).

Consequently, the following hypotheses are advanced:

Hypothesis 6: ISQ has a significant impact on PEOU.

Hypothesis 7: ISQ has a significant impact on PU.

### Information system quality and perceived value

Information system quality is described as products as well as services that meet the needs or expectations of internet users in order to complete an online transaction. The customer has easy access to particular, instant and reliable information, and accurate operation at any time and at any location. A service’s value is derived from the service provider and can also be derived from the chance for a trustworthy relationship and customer satisfaction. The quality of the information system suits the rapid consumer mentality, which has shifted to online 24-h from traditional services. The OLP decreases costs and time, which is advantageous for both parties. Previous research indicates a substantial association between ISQ and PV (Masri et al., 2020). The ISQ improves the customer relationship mentality by combining the service or product into a unified entity in an OLP. The PV depends on the user’s views of what is received and provided (Kuo et al., 2009). In an online environment, the user’s PR affects happiness and confidence in the service (Masri et al., 2020).

Based on the preceding investigations, the following hypothesis is proposed:

Hypothesis 8: ISQ has a significant impact on PV.

### Confirmation and perceived usefulness

Performance confirmation of an online platform is the belief of the first adoption that stipulates a continuous impact to drive a user’s PU, known as the belief of post-adoption associated with using the online platform (Wu et al., 2020; Zhao and Khan, 2021). Bhattacherjee (2001) further asserted that the online platform confirmation could play a significant part in determining PU for evaluating the ongoing use of an online educational system. Numerous studies have used the ECM as a conceptual framework to evaluate the utilization of online educational platforms and have found that an online system’s performance confirmation has a substantial impact on PU (Kang et al., 2009; Tsao, 2013; Hsu and Lin, 2015; Zhao and Khan, 2021). Specifically, Lu et al. (2019) highlighted a research model to analyze the relationship between cell phone advertising and consumer loyalty on the ECM. According to the results of their investigation, a substantial direct correlation was discovered between the online platform and the performance confirmation of PU (Zhao and Khan, 2021). In the light of the cited literature, the undermentioned hypothesis can be proposed.

Hypothesis 9: Confirmation has a significant impact on PU.

### Satisfaction and confirmation

The ECM indicates a substantial association between satisfaction and performance confirmation (Oliver, 1980; Zhao and Khan, 2021). Thus, a relationship between both the online platform and user satisfaction can be regarded as being established on a conceptual explanation. The ECM suggests a correlation between both the performance confirmation and user satisfaction of online platforms for assessing the purpose of continued platform utilization (Bhattacherjee, 2001; Zhao and Khan, 2021). Precisely, various research has used ECM to analyze this link for various online contexts, including social commerce, mobile commerce, and impulse purchasing (Li and Ku, 2018). Specifically, many studies discovered a considerable positive correlation between satisfaction and confirmation (Hsu and Lin, 2015; Wu et al., 2020; Zhao and Khan, 2021).

Consequently, the undermentioned hypothesis can be offered.

Hypothesis 10: Confirmation has a significant impact on satisfaction.

### Interface aesthetics and perceived value

According to a study on IAs, IAs play a significant role in identifying the satisfaction that the users encountered during the engagement process (Wang and Hsu, 2019). People’s aesthetic enjoyment influences their product inclinations positively (Yamamoto and Lambert, 1994; Wang and Hsu, 2019). Aesthetic pleasure is a desirable aesthetic response that is captured by user behavior and is favorably associated with product preferences (Graf and Landwehr, 2017). The interface design aesthetics of an online platform will affect the interpretation of functions, hedonics, and emotion (Cyr and Head, 2008; Wang and Hsu, 2019). The fundamental principle of interface design is that the interface’s aesthetic quality influences the user experience. In the majority of instances, aesthetics has a beneficial effect on actual performance when users are required to complete tasks via the UI. Aesthetically pleasing UIs result in more engaged emotional responses (e.g., users feel happy) and enhanced enjoyment while using them. User IAs influence the buying intent for consumer electronics products (Schmidt and Wolff, 2018). Consequently, it can be suggested that design aesthetics are likewise favorably related to consumers’ usage intentions (Wang and Hsu, 2019). Therefore, the undermentioned hypothesis can be proposed.

Hypothesis 11: IA has a significant impact on PV.

### Technology acceptance model and satisfaction

The link between student perception PEOU and PU of behavioral intention (BI) has been examined previously (Al-Maroof and Al-Emran, 2018; Tawafak et al., 2018b). Additionally, the integration of technology with PEOU and PU affects BI and satisfaction. The primary components of TAM plus added components of other adapted models (Tahar et al., 2013; Hone and El Said, 2016; Joo et al., 2018; Tawafak et al., 2018b). Starting with perceptions, the PEOU’s objective is for students to perceive that the model requires no work and that acquiring skills utilizing an online platform is simple (Sánchez and Hueros, 2010; Wu and Zhang, 2014; Wu and Chen, 2017). Our approach defines PEOU as the user’s assumption that future usage will be effortless (Davis, 1989; Sánchez and Hueros, 2010; Tawafak et al., 2018b). In addition, PEOU influences PU positively via TAM variables (Hong et al., 2009). Davis (1989) described PU as the belief that new technology will improve a person’s cognitive capacity. These variables can have direct effects on Satisfaction and BI, and therefore indirectly affect performance (Tawafak et al., 2018a). PU indicates the subjective system for evaluating the degree of job enhancing performance and level of progress. The PU is the direct predictor of IS behavioral intention, as sustained use is substantially impacted by PU (Lee et al., 2013; Alraimi et al., 2015). Alternately, these variables, PEOU and PU, have a substantial impact on satisfaction (Joo et al., 2018). Previous hypotheses exist on TAM participant satisfaction and initial acceptance. In light of this, this research paper regard satisfaction as the mediator between PEOU, continuing intention, and PU. Bhattacherjee (2001) utilized an ECM that utilizes acceptance variables to establish initial acceptance based on the level of satisfaction. This study focuses on motivation as an initial TAM influence on contentment. The majority of past research on this measurement has yielded consistent results. According to studies, PEOU between users on internet sites has a substantial impact on satisfaction. Additionally, PU has a substantial impact on satisfaction (Joo et al., 2018; Tawafak et al., 2018b). Built on ECM, the online platform PU has a huge effect on student contentment. This relationship is supported by the adaptations level theory, which suggests that students should only notice motivations if they have an association with an adaptive level. A previous study related to marketing has revealed that users’ levels of satisfaction are proportional to the strength of their beliefs (Zhao and Khan, 2021). For online activities, users are also required to have a positive attitude toward their conduct on internet channels, such as contentment, because digital services are deemed valuable for their alliance to discover and reveal information about the supplied services (Wu et al., 2020; Zhao and Khan, 2021). In addition, a study explored the motivating forces that influence purchasing motive for paid mobile phone applications by applying the ECM as an antecedent of satisfaction to define PU. The findings revealed a substantial association between satisfaction and PU (Hsu and Lin, 2015; Zhao and Khan, 2021). Consequently, the research hypotheses can be proposed.

Hypothesis 12: PEOU has a significant impact on satisfaction.

Hypothesis 13: PU has a significant impact on satisfaction.

### Perceived value and satisfaction

The importance of value has been constantly developed from the many studies and finding related to consumer behavior (Gallarza and Saura, 2006; Kuo et al., 2009). Prior research indicates that PV is a more accurate factor to predict intentions than contentment, commitment, or trust (Gallarza and Saura, 2006; Masri et al., 2020). Additionally, the PV of the service or product could attract new customers and result in positive outcomes for the vendors (Lai, 2004; Su et al., 2016). Our model demonstrates that user SAT is influenced by the PV of the product or service. The user’s PV impacts the user’s intention to continue using a service (Masri et al., 2020). According to the evidence presented, the undermentioned hypothesis is formulated:

Hypothesis 14: PV has a significant impact on satisfaction.

### Perceived ease of use and perceived usefulness

Perceived ease of use is the limit to which a person believes that employing particular approaches would be simple (Taufik and Hanafiah, 2019; Zhao and Khan, 2021). In addition, Legris et al. (2003) indicate the significant association between PEOU and PU through their research. PEOU is proven to have a substantial link with PU and user SAT in the setting of an online platform (Amin et al., 2014). According to an earlier study, PEOU is a significant determinant of PU in the deployment of technical items (Chen et al., 2018; Zhao and Khan, 2021). Furthermore, PU, PEOU, and perceived behavioral control have a major effect on behavioral intention to use an online service (Shin and Perdue, 2019; Zhao and Khan, 2021).

Hypothesis 15: PEOU has a significant impact on PU.

### Satisfaction and continuous intention

According to the ECM, a person’s intention to continue using an online platform depends on three factors, including their satisfaction level, post-adoption beliefs (PU), and sense of confirmation. It has been found that internet users’ SAT significantly influences their CI to utilize online platforms. According to marketing research, user SAT levels are the primary factor in determining whether they repeat certain actions (Szymanski and Henard, 2001; Lee, 2010; Zhao and Khan, 2021). As a result of the correlation between online user SAT and CI, the following research hypotheses are proposed:

Hypothesis 16: Continuous Intention has a significant impact on Satisfaction.

**FIGURE 1:**
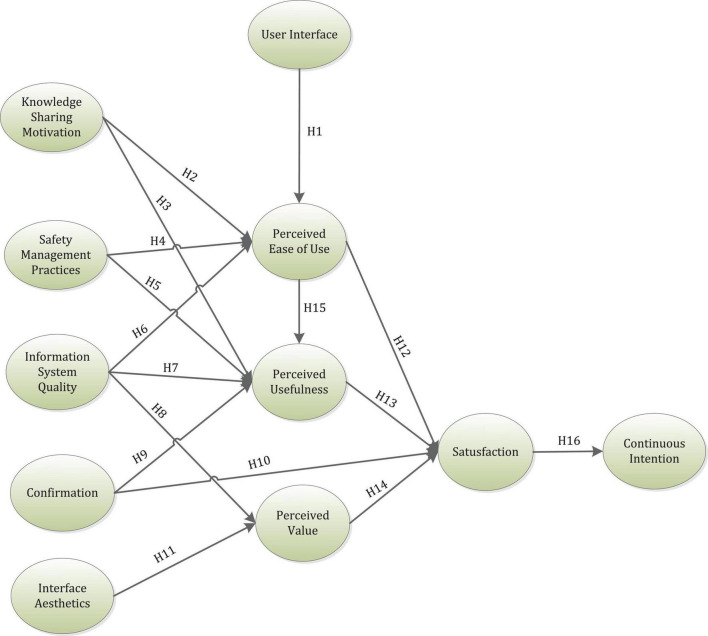
Theoretical framework.

Figure 1 represents the theoretical framework of this research study.

## Methodology

The logistics delivery sector in China provided the data for this research. The logistics couriers of China’s logistics delivery sectors were the focus of this investigation. A sample of 260 logistics couriers was targeted using a convenience sample technique. The employees of performing organizations were given a closed-ended self-administered questionnaire, and reasonable feedback was gathered that had a response rate of 90.76 percent, which is typically regarded as an ideal response rate (Comrey and Lee, 1992; MacCallum et al., 1999).

Instead of simply questioning respondents if they agree or disagree with the statements, Likert scale questions probed their level of agreeability. These questions were typically asked on a 7-point scale, with 1 denoting a strong disagreement and 7 denoting a strong agreement, with 4 denoting neutrality. The PU, Satisfaction, Confirmation, ISQ, and CI were the main factors to measure and were taken from Cheng’s (2014) study and adjusted. Additionally, the UI measurement tools were adapted and updated from Prasetyo et al.’s (2021) study. In addition, the items were taken from Wang and Hsu’s (2019) research to measure IA. Furthermore, the items adapted and modified from Xu et al. (2021)’s research were used to measure PEOU. The items recommended by Kim et al.’s (2008) research were also used to quantify PV. The items suggested by Wong et al.’s (2021) research were used to gauge SMP. Finally, the items adapted and adjusted from Hung et al. (2011) research were used to measure KSM. On 100 randomly chosen representative sample employees, a pilot test was done before the official research survey was launched. With the aid of this test, the survey questionnaires were evaluated and validated.

## Data analysis

The data analysis for this was performed using the PLS technique. The analysis consists of two phases. In the first part of the investigation, the constructs’ validity and reliability were examined. Calculating path coefficients and causal directions of the constructs constituted the second phase of the data analysis (Anderson and Gerbing, 1988; Hulland, 1999). PLS is regarded as the most effective method for calculating and maintaining the hypothesized links in complex research settings (Petter et al., 2007). In addition, PLS is appropriate for measuring the uneven distribution of findings due to the effective measurement techniques provided for addressing randomization and variable normalcy. Therefore, it is advantageous to examine innovative scientific frameworks (Chin and Newsted, 1999). To analyze data for this study, PLS was deemed to be preferable to using other structural equation models (SEM) approaches. PLS-SEM is regarded as an appropriate technique for studying complex models (Hair et al., 2014). Additionally, it is effective in determining the mediation impact of these variables (Preacher and Hayes, 2004).

### Reliability, validity, and model fit

This study’s congruent validity was determined using factor loadings, Cronbach’s alpha, rho A, CR, and AVE. The factor loading of every indicator is illustrated in Table 1. The highest factor loading is 0.930, while the lowest one is 0.791. According to the findings all the indicators’ factor loading value was higher than 0.5, which is the minimum threshold value for an indicator to signify validity threshold value of 0.5, demonstrating that each indicator has substantial validity and reliability (Xie et al., 2021). Cronbach alpha was measured to determine internal consistency, whilst CR and rho A were evaluated to determine reliability. Rho A determined the instrument’s reliability depending on the weights of its elements rather than their applied loads (Henseler et al., 2014). The appropriate Cronbach alpha (Taber, 2018) and rho A cutoff value is larger than 0.7 (Van Nguyen and Habók, 2021). According to Table 1’s findings, the Cronbach alpha, and rho_A values for all constructs were greater than 0.70. Moreover, according to the outcomes shown in Table 1, the CR values of all constructs were greater than 0.70 (Chin, 1998), showing the instrument’s internal reliability. Moreover, to determine the CR, the AVE-extracted factors for every construct were taken into account. If this attribute were greater than 0.5, the convergent validity of this construct would be high (Fornell and Larcker, 1981). Table 1 demonstrates that the AVEs for speculative construct variables in this investigation range between 0.682 and 0.802, showing substantial convergence.

**TABLE 1:** Convergent validity.

Constructs	Indicators	Factor loadings	Cronbach’s Alpha	rho_A	Composite reliability	Average Variance Extracted (AVE)
ALT	ALT1	0.874	0.918	0.918	0.918	0.736
	ALT2	0.838				
	ALT3	0.871				
	ALT4	0.847				
REC	REC1	0.842	0.896	0.896	0.896	0.683
	REC2	0.819				
	REC3	0.812				
	REC4	0.833				
REP	REP1	0.893	0.852	0.855	0.853	0.745
	REP2	0.832				
SPS	SPS1	0.866	0.884	0.885	0.884	0.718
	SPS2	0.830				
	SPS3	0.846				
SS	SS1	0.870	0.867	0.867	0.867	0.766
	SS2	0.880				
ST	ST1	0.857	0.931	0.932	0.931	0.731
	ST2	0.846				
	ST3	0.853				
	ST4	0.870				
	ST5	0.849				
SYQ	SYQ1	0.844	0.899	0.900	0.900	0.749
	SYQ2	0.897				
	SYQ3	0.855				
Information	INQ1	0.871	0.911	0.912	0.912	0.775
Quality	INQ2	0.866				
	INQ3	0.903				
SEQ	SEQ1	0.868	0.956	0.956	0.956	0.708
	SEQ2	0.840				
	SEQ3	0.853				
	SEQ4	0.815				
	SEQ5	0.829				
	SEQ6	0.850				
	SEQ7	0.844				
	SEQ8	0.843				
	SEQ9	0.828				
CI	CI1	0.823	0.888	0.890	0.889	0.727
	CI2	0.848				
	CI3	0.886				
COL	COL1	0.906	0.940	0.940	0.939	0.838
	COL2	0.909				
	COL3	0.930				
CRA	CRA1	0.888	0.909	0.910	0.909	0.770
	CRA2	0.889				
	CRA3	0.855				
DI	DI1	0.856	0.915	0.916	0.915	0.783
	DI2	0.912				
	DI3	0.886				
SI	SI1	0.884	0.917	0.917	0.917	0.786
	SI2	0.883				
	SI3	0.894				
PEOU	PEOU1	0.860	0.909	0.909	0.909	0.769
	PEOU2	0.890				
	PEOU3	0.880				
PU	PU1	0.889	0.931	0.931	0.931	0.771
	PU2	0.859				
	PU3	0.892				
	PU4	0.870				
PV	PV1	0.852	0.864	0.867	0.865	0.682
	PV2	0.842				
	PV3	0.782				
SAT	SAT1	0.894	0.942	0.942	0.942	0.802
	SAT2	0.874				
	SAT3	0.890				
	SAT4	0.924				
UI	UI1	0.791	0.920	0.922	0.921	0.700
	UI2	0.852				
	UI3	0.880				
	UI4	0.797				
	UI5	0.859				
CON	CON1	0.789	0.932	0.935	0.932	0.773
	CON2	0.876				
	CON3	0.940				
	CON4	0.906				

ALT, altruism; REC, reciprocity; REP, reputation; SPS, safety point system; SS, safety supervision; ST, safety training; SYQ, system quality; SEQ, service quality; COL, colorfulness; CRA, craftmanship; DI, diversity; SI, simplicity; UI, user interface; PEOU, perceived ease of use; PU, perceived usefulness; CON, confirmation; IA, interface aesthetics; PV, perceived value; SAT, satisfaction; CI, continuous intention.

Discriminant validity refers to the level of empirical discrepancies between two constructs. In the first investigation, discriminant validity was evaluated using the Larcker and Fornell criterion. This method uses the square root of the AVE of latent constructs (Ab Hamid et al., 2017). In this study, bootstrapping method using a confidence interval of 95% was employed to calculate the discriminant validity. According to the results indicated in Table 2, discriminant validity was achieved because neither of the paired constructs correlations’ confidence interval contained 1.

**TABLE 2:** 95% confidence interval of correlations

Parameters	Correlation	Lower bound	Upper bound
CI <-> CON	0.738	0.647	0.817
ISQ <-> CON	0.798	0.687	0.876
ISQ <-> CI	0.875	0.826	0.918
IA <-> CON	0.849	0.800	0.894
IA <-> CI	0.786	0.704	0.857
IA <-> ISQ	0.863	0.792	0.919
KSM <-> CON	0.726	0.637	0.806
KSM <-> CI	0.840	0.782	0.890
KSM <-> ISQ	0.850	0.795	0.899
KSM <-> IA	0.792	0.713	0.862
PEOU <-> CON	0.732	0.633	0.816
PEOU <-> CI	0.849	0.786	0.899
PEOU <-> ISQ	0.883	0.840	0.921
PEOU <-> IA	0.782	0.699	0.855
PEOU <-> KSM	0.841	0.778	0.896
PU <-> CON	0.758	0.668	0.835
PU <-> CI	0.828	0.758	0.890
PU <-> ISQ	0.878	0.830	0.920
PU <-> IA	0.787	0.713	0.852
PU <-> KSM	0.834	0.773	0.887
PU <-> PEOU	0.874	0.825	0.916
PV <-> CON	0.731	0.630	0.814
PV <-> CI	0.848	0.784	0.898
PV <-> ISQ	0.883	0.839	0.918
PV <-> IA	0.781	0.702	0.850
PV <-> KSM	0.845	0.790	0.893
PV <-> PEOU	0.889	0.843	0.927
PV <-> PU	0.899	0.856	0.933
SMP <-> CON	0.692	0.581	0.790
SMP <-> CI	0.806	0.718	0.880
SMP <-> ISQ	0.861	0.793	0.915
SMP <-> IA	0.779	0.685	0.857
SMP <-> KSM	0.890	0.841	0.931
SMP <-> PEOU	0.826	0.740	0.894
SMP <-> PU	0.842	0.774	0.899
SMP <-> PV	0.854	0.788	0.908
SAT <-> CON	0.733	0.638	0.814
SAT <-> CI	0.850	0.772	0.913
SAT <-> ISQ	0.876	0.826	0.917
SAT <-> IA	0.793	0.714	0.861
SAT <-> KSM	0.808	0.727	0.874
SAT <-> PEOU	0.814	0.732	0.884
SAT <-> PU	0.871	0.811	0.921
SAT <-> PV	0.828	0.738	0.899
SAT <-> SMP	0.813	0.731	0.881
UI <-> CON	0.876	0.827	0.917
UI <-> CI	0.751	0.654	0.835
UI <-> ISQ	0.829	0.715	0.905
UI <-> IA	0.898	0.858	0.932
UI <-> KSM	0.751	0.654	0.838
UI <-> PEOU	0.753	0.654	0.836
UI <-> PU	0.762	0.668	0.841
UI <-> PV	0.766	0.676	0.843
UI <-> SMP	0.738	0.627	0.831
UI <-> SAT	0.753	0.657	0.834

UI, user interface; PEOU, perceived ease of use; KSM, knowledge sharing motivation; PU, perceived usefulness; SMP, safety management practices; ISQ, information system quality; CON, confirmation; IA, interface aesthetics; PV, perceived value; SAT, satisfaction; CI, continuous intention. The value of the diagonal is the square root of AVE.

The R square is a measure of the analytical precision of the research framework that characterizes the collective impact of exogenous constructs on endogenous constructs. Although the level of acceptance varies based on the study framework, R square values are typically between 0 and 1. The larger the value, the more precise the prediction (Hair et al., 2014; Sarstedt et al., 2014). For instance, the significance of a relationship increases as the value of R square approaches 1. As a general rule, results equal to or greater than 0.6 are regarded as noteworthy, whilst values closer to 0.3 are regarded as having average prediction accuracy. Finally, values less than or equal to 0.19 are regarded as weak (Chin, 1998). According to Table 1’s results, all variables possessed values greater than 0.60, indicating a high degree of predictive precision.

This study determined the GOF utilizing the equation developed by Tenenhaus et al. (2005) to characterize the overall quality of the created model, which approximates as follows:


G⁢O⁢F=A⁢V⁢E¯⁢x⁢R2¯=0.750⁢x⁢ 0.974=0.852

As a result of the above calculation, the GOF is 0.852, which satisfies the 0.148 thresholds for significant effect size (Wetzels et al., 2009).

### Empirical results

**FIGURE 2:**
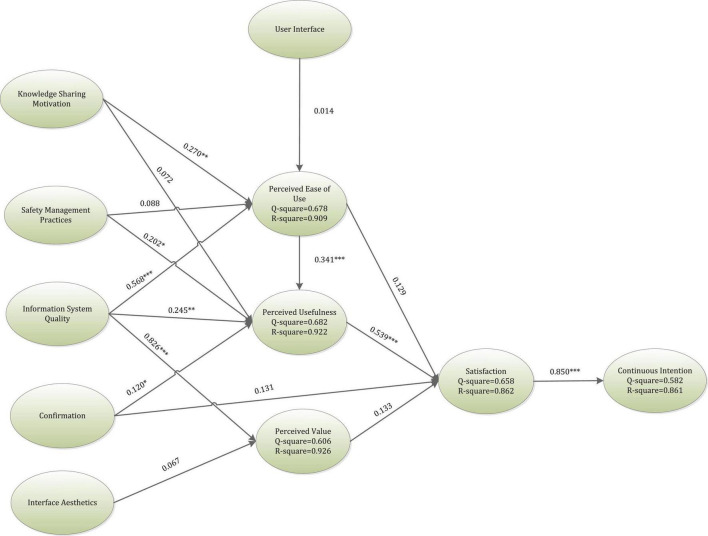
Research results. *p < 0.05, ^**^p < 0.01, ^***^p < 0.001.

Smart PLS 3.2.8 was applied for the assessment of the study framework’s route analysis. In this section, the inner model calculation was performed. To analyze the proposed hypothesis in the inner model, scholars compute the *p*-values and *t*-values. If the *p*-value is less than 0.05 or the *t*-value is more than 1.96, the suggested hypotheses are supported. Additionally, to analyze the predictive capability of the research framework, R-square analysis was employed by this research (Chen et al., 2021; Khan et al., 2021; Xu et al., 2021). Subsequently, according to the recommendation by researchers R-square is considered to be strong, moderate, or weak, if the value is around 0.67, 0.33, and 0.19 respectively (Chin, 1998). The *Q* square value is calculated by estimating the sum of square prediction errors. The values of *Q* square represent the extent to which the values are remodeled by the framework. Hence, the lesser the variance between the original and predicted values the higher will be the model’s precision (Sarstedt et al., 2014). In general, the endogenous variable’s acceptable threshold *Q* square value is greater than zero (Hair et al., 2016). Both the *R*-square and the *Q*-square values are shown in Figure 2.

As per the results of this research, as shown in Table 3 and Figure 2, UI did not have a significant association with PEOU, hence rejecting H1 (β = 0.014, *t*-value = 0.277). Furthermore, KSM was found to significantly impact PEOU, H2 (β = 0.270, *t*-value = 3.317), while having no significant effect on PU, H3 (β = 0.072, *t*-value = 0.794). Moreover, SMP was found to have no significance on PEOU H4 (β = 0.088, *t*-value = 0.721), however, SMP was discovered to be in a significant association with PU, H5 (β = 0.202, *t*-value = 2.480). In addition, ISQ was found to significantly impact PEOU, H6 (β = 0.568, *t*-value = 5.621), PU, H7 (β = 0.245, *t*-value = 2.617), and, PV, H8 (β = 0.826, *t*-value = 9.424). Moreover, CON was in a significant relationship with PU, H9 (β = 0.120, *t*-value = 2.173) while not having a significant impact on SAT, H10 (β = 0.131, *t*-value = 1.568). Furthermore, IA did not significantly impact PV, H11 (β = 0.067, *t*-value = 0.815). Also, SAT was significantly impacted by PU, H13 (β = 0.539, *t*-value = 4.968) while not having any significant impact from PEOU, H12 (β = 0.129, *t*-value = 1.572) and PV, H14 (β = 0.133, *t*-value = 1.174). Besides, PEOU was discovered to significantly impact PU, H15 (β = 0.341, *t*-value = 4.745). Finally, SAT was found to be in a significant relationship with CI, H16 (β = 0.850, *t*-value = 23.275).

**TABLE 3:** Empirical results.

Hypotheses	Path coefficient (β)	*T*-values	*P*-values
H1: UI - > PEOU	0.014	0.277	0.782
H2: KSM - > PEOU	0.270	3.317	0.001
H3: KSM - > PU	0.072	0.794	0.427
H4: SMP - > PEOU	0.088	0.721	0.471
H5: SMP - > PU	0.202	2.480	0.013
H6: ISQ - > PEOU	0.568	5.621	0.000
H7: ISQ - > PU	0.245	2.617	0.009
H8: ISQ - > PV	0.826	9.424	0.000
H9: CON - > PU	0.120	2.173	0.030
H10: CON - > SAT	0.131	1.568	0.117
H11: IA - > PV	0.067	0.815	0.415
H12: PEOU - > SAT	0.129	1.572	0.116
H13: PU - > SAT	0.539	4.968	0.000
H14: PV - > SAT	0.133	1.174	0.240
H15: PEOU - > PU	0.341	4.745	0.000
H16: SAT - > CI	0.850	23.275	0.000

UI, user interface; PEOU, perceived ease of use; KSM, knowledge sharing motivation; PU, perceived usefulness; SMP, safety management practices; ISQ, information system quality; CON, confirmation; IA, interface aesthetics; PV, perceived value; SAT, satisfaction; CI, continuous intention.

## Conclusion and discussions

The current study integrated the Extended Technology Acceptance Model and included ISQ, KSM, ECM, IA, UI, and PV to evaluate the logistics couriers’ experience while using an Online logistics platform. This research examines the relationships of KSM, SMP, and ISQ on TAM’s PU and PEOU. In addition, it explores the relationship of UI on PEOU. Furthermore, to explore the impact of ECM, it examines the impact of confirmation on PU and satisfaction. Moreover, the present study investigates the impact of ISQ, and IA on PV. Additionally, this study measures the impacts of PU, PEOU, and PV on satisfaction. Also, this research explores the influence of PU on PEOU. Finally, this research explores the impact of logistics couriers’ satisfaction on continuous intention.

According to the findings of this study, UI did not show a significant relationship with PEOU. The present findings differ slightly from those of earlier research published by Prasetyo et al. (2021). The focus of Prasetyo et al.’s (2021) research was to identify the characteristics influencing students’ adoption of an educational platform during the COVID-19 outbreak. Extended TAM was used in their research, while SEM was used to examine various other concepts, including PEOU, PU, UI, intents, actual usage, System Quality, and information quality. According to the findings, PEOU was found to have the highest impact on actual use, with UI and quality of the system toward PEOU coming in second and third, respectively, before intents and actual usage. The students’ level of acceptance takes the SQ, IQ, EOU, and UI into account, resulting in a positive behavior intent for actual use (Prasetyo et al., 2021).

Moreover, KSM, in its association with TAM, was found to have a substantial influence on PEOU but no effect on PU. The findings of the current study were fairly comparable to those of earlier research by Hung et al. (2011). The objective of Hung et al.’s (2011) research was to determine whether R&D professionals would be willing for knowledge-sharing using an EKR. Their investigation utilized TAM to determine the impact of extrinsic and intrinsic motives on R&D employees’ acceptance of an EKR for knowledge-sharing. Empirical findings are obtained through survey conduction in Taiwan. Reciprocation and Reputation were identified as two essential precursors to PU and PEOU, whereas altruism was identified as an antecedent variable to PEOU. The most powerful aspect of PU was reputation, although reciprocation was another influential variable (Hung et al., 2011).

Furthermore, SMP impact according to TAM showed that SMP had no major impact on PEOU, while SMP was shown to be in a substantial relationship with PU. The findings of the current study are equivalent to those of a study by Wong et al. (2021). As per the research of Wong et al. (2021), the majority of construction casualties are due to falls from heights, which are caused by not using PPE. Their objective of the research was to propose a research model that blends the TAM, SMP, and safety awareness to explain construction workers’ acceptance of PPE. The influence of these constructs on PPE acceptance was examined using mediation and SEM analysis. The results suggested that the SMP were significant in developing attitudes about PPE use through the mediation of PU, PEOU and safety recognition.

Additionally, it was discovered that ISQ had a substantial impact on both TAM parameters, both PU and PEOU. The findings are consistent with research done by Kim et al. (2008). Kim et al.’s (2008) research indicated to increase organizational effectiveness and strategic competitiveness, hospitality organizations should invest in IT. In their work, the authors adopted TAM to examine the relationship between antecedents like PV and ISQ and users’ approval of HFOSs. Empirical results showed that all factors are substantial. To improve the model, their research was able to determine the acceptance of HFOSs from the viewpoint of hotel front-line staff using the external factors of ISQ and PV (Kim et al., 2008). Additionally, the present study indicates that ISQ is substantially associated with PV. The finding is consistent with a previous study performed by Masri et al. (2020). Their research centered on the creation of e-travel information that permits consumers to arrange trips without regard to space or time constraints. In the context of e-tourism, their research offered a model for the establishment of relationship quality, ISQ, PV, and CI. The study’s location is Taiwan. The Results indicated a favorable association between ISQ and customer contentment, trustworthiness, and customer intent to continue (Masri et al., 2020).

Moreover, the latest research also examined the effect of ECM. According to the outcomes, CON had a significant association with PU, although SAT did not. The current study’s findings can be correlated to those of earlier research published by Mamun et al. (2020). Their research creates and evaluates an expanded ECM framework in order to investigate the IT retention behavior in the work and in life. The suggested model replaces the perceived advantage for PUs, which featured heavily in earlier ECM models but fails to account for the non-utilitarian benefits of IT. In addition, the latest design captures emotional deviances and illustrates consequences in the continuous dependent variable (Mamun et al., 2020).

Moreover, IA had no significant effect on PV. The outcome can be paralleled to research made by Wang and Hsu (2019). This research entrenched a new product aesthetics division, classified it into aesthetics in form of product and IA, and suggested a new intellectual model check the effect of product form aesthetics and IA on purchase intention and PV. In this research, smartwatches were the subject and PLS was implemented to check the intellectual model. The research findings proposed that PV must mediate the product from aesthetics and IA (Wang and Hsu, 2019). Also, SAT was substantially affected by PU, whereas PV and PEOU have no substantial effects. These findings are paralleled earlier research (Tawafak et al., 2018b; Masri et al., 2020; Zhao and Khan, 2021). Besides, it was analyzed that PEOU significantly impacts PU, and the results are comparable to research conducted by Zhao and Khan (2021). Finally, it was discovered that SAT is substantially related to CI, The result was almost similar to previous studies (Masri et al., 2020; Zhao and Khan, 2021).

## Theoretical implications

The research study based its theoretical research model based on an extended TAM, ECM, SMP including SOPS (safety-offense points), SS (safety supervision) and ST (safety training), ISQ concept including information quality, system quality, and service quality, KSM, IA including four dimensions simplicity, diversity, colorfulness and craftmanship, and PV. The findings of this study have several ramifications. First, this study extends the previously researched ISQ (DeLone and McLean, 2003), and its findings contribute to the research on ISQ by investigating information quality, system quality, and service quality as a unified entity (DeLone and McLean, 2003; Masri et al., 2020).

Theoretically, this study expands the notion of SMP by incorporating it with TAM to evaluate the logistics couriers’ experience while utilizing OLPs. This study significantly expanded the TAM literature regarding logistics courier safety. However, how to properly build a positive attitude among logistics couriers by ST is a crucial problem in motorcyclists’ safety that demands extensive research (Loosemore and Malouf, 2019; Wong et al., 2021). By evaluating the influence of ST, SOPS, and SS as a single variable on PEOU, and PU, this study successfully addressed a research gap in the literature.

In addition, this study contributed to OLP literature by illuminating the effects of KSM on OLP acceptability. This research specifically suggests the presence of three incentives, namely altruism, reciprocation, and reputation based on SET. According to Davenport and Prusak (1998), these are also hypothesized to stand out in the market for learning. This research paper thinks that more research is needed to clarify the connections between TAM and user sharing motivations. Research of other sharing reasons, in particular, was skipped. Further research is also required to determine if other KSM, such as knowledge self-efficacy, rewards, and knowledge growth, influence TAM (Hung et al., 2011).

According to the findings of this study, UI did not show a significant relationship with PEOU. The present findings differ slightly from those of earlier research published by Prasetyo et al. (2021). The reason for demonstrating a different result can be the target sample, which in this study were the logistics couriers, unlike the previously conducted studies. Moreover, the location of the research can also be a factor in signifying comparatively differential results from the past. For instance, this research was conducted in China, which is an emerging economy, hence, the results can not be compared to the studies conducted in developed economies. Consequently, as per the findings of this research, all scholars, administrators, and policymakers are proposed to prioritize a user-friendly OLP UI to support the OLP’s use for knowledge-sharing and make it easier for individuals to share their expertise. Mature innovation technologies have reduced UI issues because consumers now possess the required knowledge and confidence (Wu and Wang, 2005). Therefore, as consumers gain familiarity, the influence of usability will diminish over time (Hung et al., 2011). Additionally, additional research is required to examine the OLP’s implementation period.

## Practical implications

Practical implications for KSM, UI, PV, IA, SMP, ISQ, and logistics couriers CI are significant for OLPs managers. To increase IS reliability in the OLP, managers must modernize the operational process architecture and delivery service procedure to meet the requirements of real-time users. In addition, they need hardware and software with an efficient IS to prevent technical issues and transaction overload (Hadji and Degoulet, 2016). These data can be utilized by practitioners and managers as guides for the development of online portals, operations, and user assistance. The assessment of ISQ can improve goods and services, enabling managers and enterprises to produce superior commodities in the OLP. This research study’s observations not only reflect a perspective on ISQ improvement but also on enhancing user relationship value via the OLP provider’s application of customer contentment. In addition, the study proved the significantly positive impact of PV on users’ CI via customer satisfaction, which increases the long-term success of adopting the new program. This may show that improving ISQ is not only beneficial for users but also facilitates the development of user interactions in an OLP setting (Masri et al., 2020). This research demonstrates that SOPS along with ST played a significant effect on logistics couriers’ acceptance of safety. Therefore, the management of OLPs should develop an efficient SOPS. When this method is implemented in the OLP business, logistics couriers who do not utilize SMP at work will receive SOPS. Logistics couriers must complete additional ST sessions when their Points reach a certain threshold. Non-completion of the additional courses shall result in disciplinary sanctions, such as the removal of safety incentives. Traditional ST’s performance in the building industry is inadequate (Gao and Wu, 2019). ST equipped with VR displays can enhance logistics couriers’ safety development and self-efficacy. Therefore, The senior management of OLPs should combine VR technology with ST to improve ST’s efficacy (Wong et al., 2021). Moreover, the results indicate that altruism, reputation, and reciprocity are three precursors of the PEOU. Therefore, businesses should build a culture of knowledge-sharing before implementing the OLP to foster altruism and reciprocity. All those who believe in reciprocation anticipate receiving others’ favors in the future; therefore, they exchange ideas for each other’s welfare. This study concludes that the incentive of reciprocity is essential for sustained information sharing. If organizations hire talented employees and manage them well, knowledge generosity can be encouraged and an atmosphere of generosity could be developed; nevertheless, people will become disheartened if the employer places increasing demands on their time and energy. Additionally, creating a suitable reputation system for the EKR will assist staff in developing their professional identities. In these situations, people can exert themselves more and feel more confident (Hung et al., 2011).

Additionally, this study also demonstrated that IA had no significant effect on PV. The outcome can be paralleled to research made by Wang and Hsu (2019). Previous research focused on the product’s IA, while this research was based on the examination of IA of OLPs. Hence, this research study is illustrating different findings related to the IA as compared to some of the previous research work. Likewise, this research study also examined the effect of ECM. According to the outcomes, the CON part of the ECM did not have a significant impact on SAT. In terms of this result, this research findings are somewhat different than earlier research conducted by Mamun et al. (2020). The research findings are different from the previous research because this research targeted OLPs and logistics couriers as compared to some of the previous research that used office workers’ acceptance of technology.

## Limitations and future research directions

During the execution of this investigation, the researchers came into a few challenges. Consequently, this work provides limitations and possibilities for future research. This study employed KSM about TAM. The behaviors of knowledge-sharing and knowledge-seeking are separate. Future research could examine the impact of employees’ knowledge-seeking incentives on their adoption of the OLP for knowledge-seeking to better comprehend the behavioral intention to use the OLP. Although the results demonstrate that ISQ influences users’ acceptance and use of technology beliefs OLPs, it is imperative to note that other factors also may play a significant impact on users’ technology acceptance. Examples of such aspects include computer self-assertiveness (Ong and Lai, 2006), inventiveness (Lu et al., 2005), and experience of using a computer (Zain et al., 2005). Future research should expand the search for causes that influence user views regarding technological acceptance. In addition, the study data were obtained in China, a developing nation, so it is recommended that future researchers use the research framework in advanced economies to gain new insights. In addition, future academics will be able to compare emerging and industrialized economies.

## Data availability statement

The raw data supporting the conclusions of this article will be made available by the authors, without undue reservation.

## Author contributions

QZ and CL: conceptualization, methodology, formal analysis, investigation, and visualization. SB: validation. QZ, CL, and SB: writing – original draft, review and editing. All authors read and agreed to the published version of the manuscript.
